# Pepper SBP-box transcription factor, *CaSBP13*, plays a negatively role in drought response

**DOI:** 10.3389/fpls.2024.1412685

**Published:** 2024-07-12

**Authors:** Huai-Xia Zhang, Yuan Zhang, Bo-Wen Zhang

**Affiliations:** College of Horticulture and Landscape Architecture, Henan Institute of Science and Technology, Xinxiang, Henan, China

**Keywords:** pepper, CaSBP13, drought, Nicotiana benthamiana, ABA signaling pathway

## Abstract

The SBP-box gene significantly influences plant growth, development, and stress responses, yet its function in pepper plants during drought stress remains unexplored. Using virus-induced gene silencing and overexpression strategies, we examined the role of *CaSBP13* during drought stress in plants. The results revealed that the expression of *CaSBP13* can be induced by drought stress. Silencing of *CaSBP13* in pepper notably boosted drought resistance, as evident by decreased active oxygen levels. Furthermore, the water loss rate, relative electrical conductivity, malondialdehyde content, and stomatal density were reduced in CaSBP13-silenced plants compared to controls. In contrast, *CaSBP13* overexpression in *Nicotiana benthamiana* decreased drought tolerance with elevated reactive oxygen levels and stomatal density. Additionally, ABA signaling pathway genes (*CaPP2C*, *CaAREB*) exhibited reduced expression levels in CaSBP13-silenced plants post drought stress, as compared to control plants. On the contrary, *CaPYL9* and *CaSNRK2.4* showed heightened expression in CaSBP13-sienced plants under the same conditions. However, a converse trend for *NbAREB*, *NbSNRK2.4*, and *NbPYL9* was observed post-four day drought in CaSBP13-overexpression plants. These findings suggest that *CaSBP13* negatively regulates drought tolerance in pepper, potentially via ROS and ABA signaling pathways.

## Introduction

With global climate shifts and expanding human activity, demand for water resources escalates, leading to widespread drought disasters globally. Droughts persist for lengthy periods and exert substantial influence on agricultural productivity (Vicente-Serrano et al., 2022). The absence of moisture impairs plant development across all stages. It diminishes seed germination, planting vigor, canopy development, root elongation, and subsequently triggers yield reduction or plant mortality (Abbas et al., 2023). Pepper, a highly valued vegetable crop, due to its shallowly rooted system with reduced regeneration capacity after root damage, making it especially prone to drought stress. Over time, vegetables have evolution multi-tiered defensive strategies against drought to sustain growth and metabolic processes (Abbas et al., 2023). Examples of physiological and biochemical alterations include the activation of stress responsive genes, which predominantly controlled by transcription factors (Thirumalaikumar et al., 2018; Zhang et al., 2023). For instance, the transcription factor *AtJUB1* positively modulates stress response mechanisms in tomato under drought conditions. It has been demonstrated that *AtJUB1* triggers *DREB2A* gene expression, a crucial transcriptional regulator of drought tolerance, and the *DELLA* genes *GAI* and *RGL1* (Thirumalaikumar et al., 2018). *OsERF71* enhances drought tolerance through augmenting the expression of ABA signaling and proline biosynthesis-correlated genes during drought conditions (Li et al., 2018). Lastly, in dicot *DcPIF3* attenuates drought-driven ROS generation, upregulating ABA biosynthetic genes expression to augur increased cellular ABA concentration and thereby boosting drought resistance in carrot (*Daucus carota* L.) (Wang et al., 2022).

The SBP-box gene, a plant-specific transcription factor, possesses a well-conserved DNA-binding domain, the SBP domain (Klein et al., 1996; Cardon et al., 1999). Comprising approximately 76 amino acid residues, this domain participates in DNA binding and nuclear localization, inclusive of two zinc-binding sites (Yamasaki et al., 2004). Initially identified in *Amirrhinum majus*, the gene is composed of two subunits, *AmSBP1* and *AmSBP2*, which interact with SQUAMOSA’s promoter to influence early floral development (Klein et al., 1996). Associated research indicates these genes could be pivotal in floral morphogenesis and developmental regulatory networks (Cui et al., 2020; Jeyakumar et al., 2020; Yang et al., 2023; Wei et al., 2024). Additionally, their potential role in abiotic stress adaptation has recently gained attention. In *alfalfa*, miR156’s pivotal role in enhanced drought resilience was observed via repression of *SPL13* and up-regulation of *WD40-1*, similarly, stimulatory action of miR156 increased drought susceptibility (Arshad et al., 2017; Feyissa et al., 2019). Equally, *OsSPL10* regulator rice’s response to salt stress, affecting both *OsNAC2* expression and drought-induced ROS production (Lan et al., 2019; Li et al., 2023). The SBP-box gene (*CnSBP7/9//10/14/17/19*) of *Chrysanthemum nankingense* also reacts to drought stress (Lan et al., 2019). In Sweet Orange (*Citrus sinensis*), 15 genes showed response variations under low/high temperature and salt conditions (Song et al., 2021). Notably, the *miR156/SPL* module stimulates *MdWRKY100* expression enhancing salt tolerance in apple (Hu et al., 2023). Furthermore, in pigeon pea (*Cajanus cajan*) under drought stress, increased levels of *CcSPL2.1*, *3*, *13A* were observed while prominent up-regulation of *CcSPL14* and *15* was noted in salt-susceptible cultivars (Shaheen et al., 2024). Enhanced *TaSPL6* expressions decrease drought tolerance, conversely, decreased *TaSPL6* expressions improved drought resistance in wheat (Zhao et al., 2024). Enhanced drought resistance was observed in *Arabidopsis* overexpressing the ‘SiJiMi’ mango gene, *MiSPL3a/b* (Zhu et al., 2024).

However, according to our knowledge, no research exists elucidating the function of pepper SBP-box gene in drought stress tolerance. Hence, this study examined the contribution of *CaSBP13* gene (Accession No. Capana10g002379) in pepper under drought stress. Results indicate that *CaSBP13* suppresses pepper’s drought stress response, potentially via ROS and ABA signaling pathways.

## Materials and methods

### Plant materials and growth conditions

The plant materials used in this study consisted of the pepper cultivar AA3 and (*Nicotiana benthamiana*) *N. benthamiana*, sourced from the Horticultural Landscape Architecture faculty of Henan Institute of Science and Technology, Xinxiang 453003, China. Pepper was grown under controlled conditions (16/8 hour photoperiod, 22°C daytime/18°C nighttime temperature, and 80% humidity). *N. benthamiana* was cultivated at optimized conditions of 16/8 hour photoperiod, 25°C/18°C day/night temperature, and 60% humidity.

### Virus-induced gene silencing of *CaSBP13* in pepper

The *CaSBP13* gene of pepper was silenced following the VIGS protocol described by Wang in 2013 (Wang, 2013). A 310bp fragment from the pepper *CaSBP13* gene was amplified using specific primers, which were verified in both NCBI (http://www.ncbi.nlm.nih.gov/tools/primer-blast/index.cgi?LINK_LOC=BlastHome) and pepper genome databases (http://peppergenome.snu.ac.kr/) (**Supplementary Table 1**). The PCR product and TRV2 plasmid were cleaved with *BamH*I and *Kpn*I restriction enzymes, followed by linkage using the T4-DNA ligase (Trans Gen Biotech, Beijing, China). The recombinant vector was sequenced at Sangon Biotech Company (Shanghai, China). The resultant vectors, i.e., TRV2:*CaSBP13*, TRV2: *CaPDS* (phytoenedesaturase, positive control), TRV2:*00* (negative control), and TRV1, were transduced into *Agrobacterium tumefaciens* strain GV3101 via freeze-thaw technique.

A total of 400 pepper seedlings at the second true leaf phase (40 days post-germination) were utilized for *CaSBP13* gene silencing as per the protocol outlined by Zhang et al. (2013). All seedlings exposed to infection were maintained in a controlled environmental growth chamber as per Wang’s specifications (Wang, 2013). Photo-bleaching noticed in TRV2:*CaPDS* positive subjects prompted collection of leaf samples from both the CaSBP13-silenced (TRV2:*CaSBP13*) and control (TRV2:00) plants for assessing efficiency of gene silencing.

### Overexpression of CaSBP13 in *N. benthamiana*

The full-length sequence of *CaSBP13* lacking its termination codon was amplified and cloned into a pVBG2307:GFP vector at the *Xba*I and *Kpn*I restriction sites to form the pVBG2307:CaSBP13:GFP construct (**Supplementary Table 1**). Confirmation was achieved through sequence analysis by Sangon-Biotech Company (Shanghai, China). Subsequently, the recombinant pVBG2307:CaSBP13:GFP vector was utilized for *CaSBP13* overexpression in *N. benthamiana*. Transgenic *N. benthamiana* overexpressing *CaSBP13* were produced via *Agrobacterium tumefaciens*-mediated leaf disc transformation (Oh et al., 2005). We identified three kanamycin-resistant *CaSBP13* transformants with RNA confirmation. T1 progeny were derived from T0 plants, and T2 progeny from T1 plants. Herein, we chose T3 progeny for further study.

### Stress treatments and samples collection

To assess *CaSBP13* transcript levels in peppers exposed to drought stress, a total of 200 seedlings with six to eight developed leaves were harvested from a substrate composed of matrix, vermiculite, and perlite (in a 3:1:1 ratio), subsequently reared in 1/2 Hoagland’s solution, and exposed to 20% Polyethylene glycol (PEG6000) after three days. A control group remained in 1/2 Hoagland’s solution only (Li et al., 2022). Leaves from 6-8 plants were collected at time 0h, 3 h, 6 h, 12 h, 24 h, and preserved at −80°C.

For ABA stress, a total of 200 seedlings were treated with 20μM ABA employing the method outlined by Yin et al. (2014). Aqueous solution consisting of 0.5% tween and 0.1% ethanol served as the control for ABA exposure. Leaves from 6-8 plants were harvested at 0, 3, 6, 12, 24, and 48 hours, promptly frozen with liquid nitrogen and preserved at -80°C.

The drought experiments of CaSBP13-silenced and CaSBP13-overexpressing plants were adapted from Liu’s methodology, with minor modifications (Liu et al., 2017). For CaSBP13-silenced plant’s drought treatment required consistent exposure of all peppers in identical environmental conditions. A single deep watering occurred one month prior to the drought treatment, followed by equal watering every three days. After the last watering three days was considered the initiation of drought stress and was denoted as day 0. Samples from 6-8 plants were collected and preserved at −80°C at time intervals of 0, 1, 2, 3, 4 days post-drought.

For CaSBP13-overexpressing plants under drought stress, the same procedure was employed with minor alterations (672 seedlings were used for this experiment): A single deep watering was provided one month before drought, followed by equal watering every four days. The fourth day post the last watering marked day 0 of drought stress. Samples from 6-8 plants were collected and stored at −80°C after post-drought at 0, 2, 4 days intervals.

The detection of seed germination rate and root length for CaSBP13-overexpression plants is according to the method described by Ma et al. (2011). The seeds are immersed in ABA solutions at 0g/L, 0.1g/L, 0.5g/L, and 1g/L concentration gradients for 24 hours, and then placed on double-layered filter paper moistened with deionized water in a culture dish. The dish is maintained in a 25°C light incubator with 16 hours of day and 8 hours of night, with regular ventilation for 10 minutes and added fresh water to maintain moisture daily. Germination counts are recorded on the third, fifth, and tenth days. The seeds of CaSBP13-overexpression plants that germinated without any treatment during the same period undergo identical ABA treatment and cultivation methods. Root length is measured after 10 days treatment.

### RNA extraction and real-time quantitative PCR

Total RNA was isolated using the Takara MiniBEST Plant RNA Extraction Kit per manufacturer’s guidelines (Takara, Dalian, China). The first strand synthesis was accomplished with the Prime Script Kit (Takara, Dalian, China). The resultant cDNA solution was standardized to 50 ng/L and utilized for real-time quantitative (qPCR) analysis.

The Bio-Rad iCycler thermocycler (Hercules, CA, USA) performed real-time qPCR as per Zhang et al. (2020) methodology (Zhang et al., 2020). This included a pre-denaturation phase at 95°C for 1 minute, followed by 40 cycles of denaturation (95°C, 10 seconds), annealing (56°C, 30 seconds), and elongation (72°C, 30s). Fluorescence quantification was conducted at each cycle’s completion via post-PCR melting curve analysis ranging from 56 to 95°C for assessing primer specificity. Pepper ubiquitin-binding protein gene (*CaUBI3*) served as a reference while *Nicotiana benthamiana* actin gene, *Nbactin-97*, functioned as a control in *Nicotiana benthamiana* experiments (Du et al., 2015; Zhang et al., 2016). All primer pairs were verified through NCBI Primer BLAST. The specificities of these primers are provided in **Supplementary Table 1**. Gene expression was quantified utilizing the 2^-△△CT^ method (Schmittgen and Livak, 2008).

### Physiological indicators measurement

The determination of malondialdehyde (MDA), relative electrical conductivity, and relative water content (RWC) was performed per Zhang et al. (2018) and Pan et al. (2012). Water loss calculations followed Ma et al. (2021) protocol. Chlorophyll content quantification used an approach detailed by Arkus et al. (2005). The activities of peroxidase (POD), superoxide dismutase (SOD), and catalase (CAT) were examined per Zhang et al. (2018) and Stewart and Bewley (1980).

The methods of DAB and NBT staining for hydrogen peroxide (H_2_O_2_) and oxygen (O_2_^-^) radical analysis were adapted from Kim et al. (2012) and Thordal-Christensen et al. (1997). Their stain areas were quantified based on Sekulska-nalewajko et al. (2016). H_2_O_2_ content was determined using Liu et al. (2010). Superoxide anion (O_2_^-^) detection is executed as per Solarbio Superoxide Anion kit instructions (Solarbio, Beijing, China).

Scanning electron microscopy (SEM), FEI Quanta 200 (USA), allowed a high resolution investigation of stomata morphologies. The imaging was quantified using Image J software from the National Institutes of Health. The stomatal apertures was also detected using SEM. The stomatal aperture size includes the length and width of pores, with the pore length determined in this test as the dumbbell-shape aperturae. Pore width denotes the maximum value perpendicular to the dumbbell-shape aperturae.

### Statistical analysis

Statistical evaluation was executed using SPSS 22.0 software. One-way ANOVA evaluated treatment variations. Significant distinctions were established at *P* ≤ 0.05 and *P* ≤0.01 utilizing Tukey’s *post hoc* test. Data are showcased as mean ± SD (standard deviation). All experiments were independently conducted, with a minimum of three biological replicates.

## Results

### Expression of the *CaSBP13* gene in pepper under drought and ABA stress

To explore the role of *CaSBP13* in drought stress, we evaluated its transcriptional profile post-stressor. As revealed in **Supplementary Figure 1**, the *CaSBP13* gene displays significant increases post-drought stress, peaking at 6 hours post-stress. Further, the expression of *CaSBP13* fluctuates, notably attenuating at 12h after ABA treatment. These data suggest a plausible involvement of *CaSBP13* in drought stress and ABA treatment response.

### Silencing the *CaSBP13* gene improved pepper plant resilience to drought stress.

To further investigate the function of *CaSBP13* under drought stress, it was silenced utilizing the virus-induced gene silencing method (Wang, 2013). Here, a positive control vector, TRV2:*CaPDS*, silenced the *CaPDS* gene, resulting in photo-bleached leaves. Meanwhile, TRV2:*00* served as a negative control. At the appearance of photo-bleached leaves in TRV2:*CaPDS* plants, the silencing efficacy of TRV2:*CaSBP13* and TRV2:*00* was examined (see **Supplementary Figure 2**). It shows morphologically identical states between CaSBP13-silenced (TRV2:*CaSBP13*) and control (TRV2:*00*) plants under normal conditions, attesting to over 94% silencing efficiency. Consequently, both CaSBP13-silenced and control plants were selected for further analysis.

**Figure 1:**
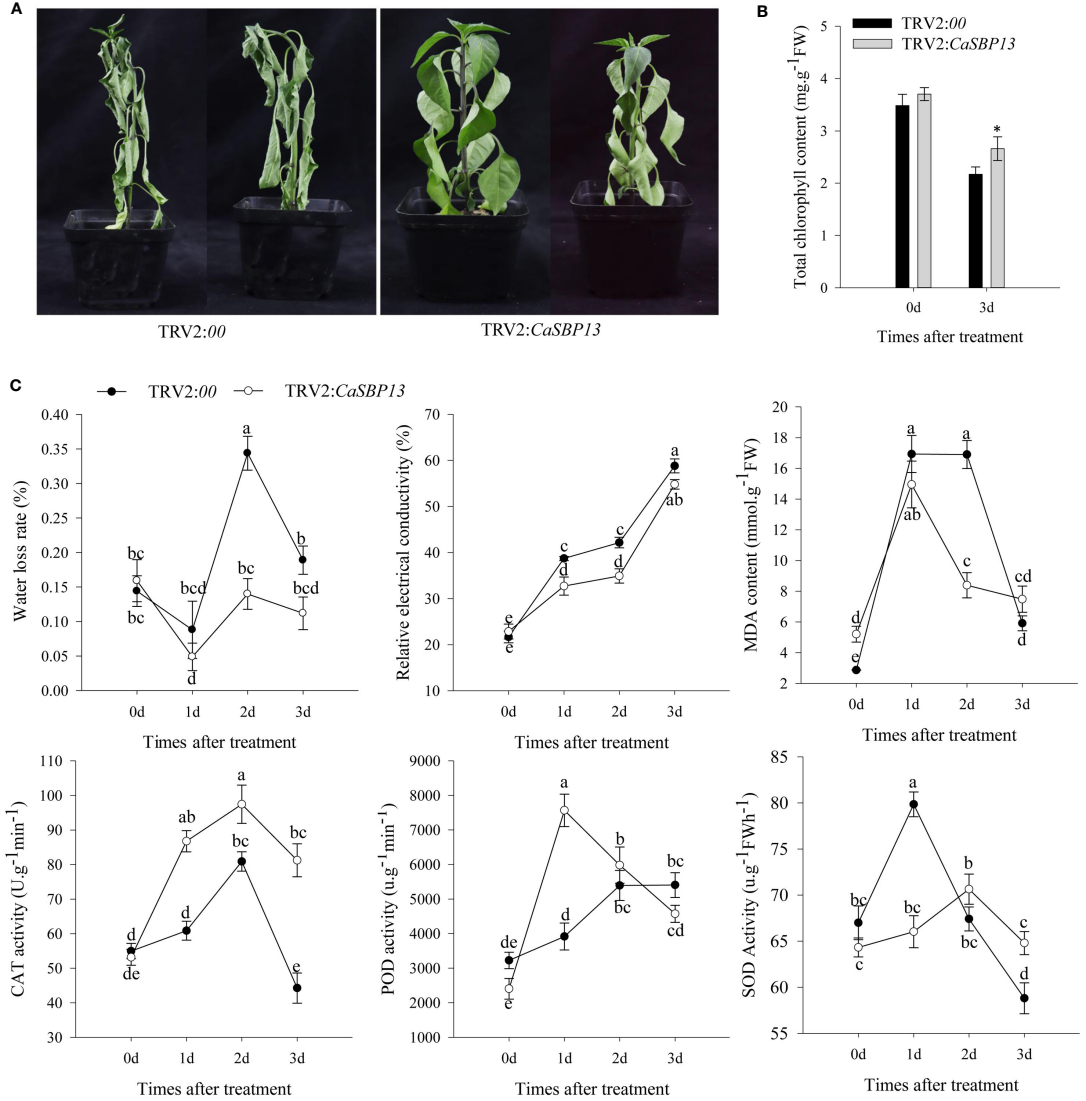
Identifying drought tolerance of CaSBP13-silenced plants. **(A)** Phenotypes of the CaSBP13-silenced and control plants after three days of drought stress. **(B)** The total chlorophyll content of CaSBP13-silenced and control plants after three days of drought stress. **(C)** The water loss rate, relative electrical conductivity, malondialdehyde (MDA) content, peroxidase (POD), catalase (CAT) and superoxide dismutase (SOD) activities of CaSBP13-silenced and control plants after drought stress. d: day. One-way ANOVA was employed to examine the differences between treatments, significant variances were identified via Tukey’s *post hoc* test. *represent significant differences at *P* ≤ 0.05. Bars with different letters indicate significant differences at *P* ≤ 0.05. Mean values and SDs for three replicates are shown.

After three days of drought stress, a wilt was observed in lower leaves of CaSBP13-silenced plants. In contrast, the leaves of control plants displayed almost complete wilt (**Figure 1A**). Upon drought stress, both CaSBP13-silenced and control plants exhibited a decrease in chlorophyll content; however, the former had significantly greater amounts than the latter (**Figure 1B**). Additionally, the water loss rate of CaSBP13-silenced plants was distinctly lower than control plants at two days of drought, exhibited a complex pattern (**Figure 1C**). Similarly, the relative electrical conductivity of CaSBP13-silenced and control plants trended upward, yet control plants consistently surpassed CaSBP13-silenced plants, especially on Days 1 and 2 (**Figure 1C**). The MDA content of both plant types showed an ascending-descending trajectory, with control plants exhibiting higher levels on Days 1 and 2 (**Figure 1C**). POD, CAT, and SOD activity in CaSBP13-silenced and control plants initially rose, then declined, with CAT activity being significantly higher in CaSBP13-silenced plants than control ones. POD activity was notably higher in CaSBP13-silenced plants than control plants at day 1, while SOD activity followed the opposite trend compared to POD activity (**Figure 1C**). These findings indicate enhanced drought tolerance in CaSBP13-silenced plants.

**Figure 2:**
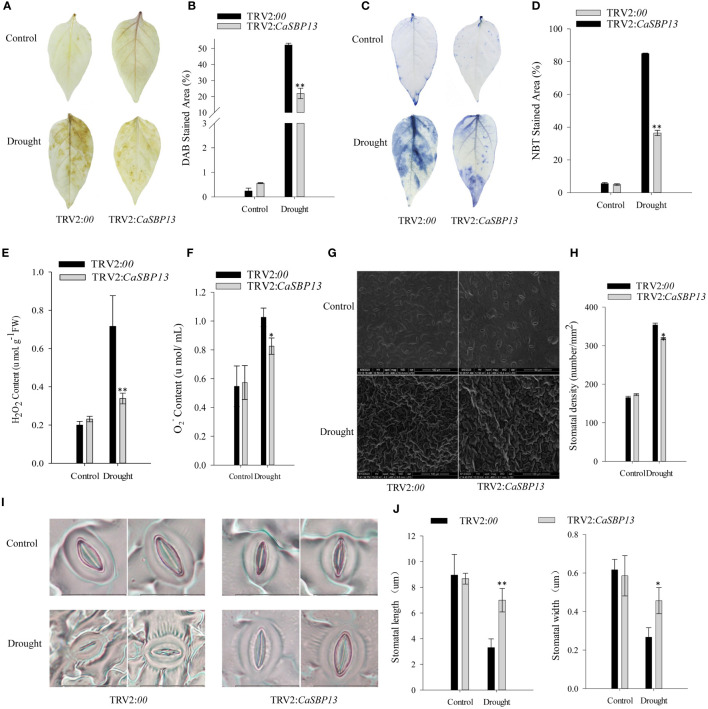
DAB and NBT staining of CaSBP13-silenced and control plants, coupled with quantification of stomatal density and apertures. **(A)** DAB staining in leaves of CaSBP13-silenced and control plants after four days of drought stress. **(B)** DAB stained area of CaSBP13-silenced and control plants after four days of drought stress. **(C)** NBT staining in leaves of CaSBP13-silenced and control plants after four days of drought stress. **(D)** NBT stained area of CaSBP13-silenced and control plants after four days of drought stress. **(E)** The H_2_O_2_ content of CaSBP13-silenced and control plants after four days of drought stress. **(F)** The O_2_^-^ content of CaSBP13-silenced and control plants after four days of drought stress. **(G, H)** stomatal density assessment. Scale bar, 100 µm. **(I, J)** The morphological features and length/width of stomata. Scale bar, 20 µm. One-way ANOVA was employed to examine the differences between treatments, significant variances were identified via Tukey’s *post hoc* test. * and ** represent significant differences at *P* ≤ 0.05 and *P* ≤0.01 respectively. Mean values and SDs for three replicates are shown.

Furthermore, to evaluate the increase of reactive oxygen species (ROS) in both CaSBP13-silenced and control plants under drought stress, hydrogen peroxide (H_2_O_2_) and superoxide anion (O_2_^-^) levels were measured using DAB and NBT staining (**Figures 2A–D**). After four days of drought, significant differences were observed between control and CaSBP13-silenced plants for leaf area displaying DAB and NBT staining (**Figures 2A–D**), suggesting higher H_2_O_2_ and O_2_^-^ in controls (**Figures 2E, F**). Also, considering relative stomatal density, control plants had a higher number than CaSBP13-silenced plants (**Figures 2G, H**). Besides, post drought stress four days, stomatal length and width decrease. However, these parameters in the CaSBP13-silenced plants are notably larger than those of the control plants (**Figures 2I, J**). This indicated lower ROS accumulation in CaSBP13-silenced leaves compared to controls.

**Figure 3:**
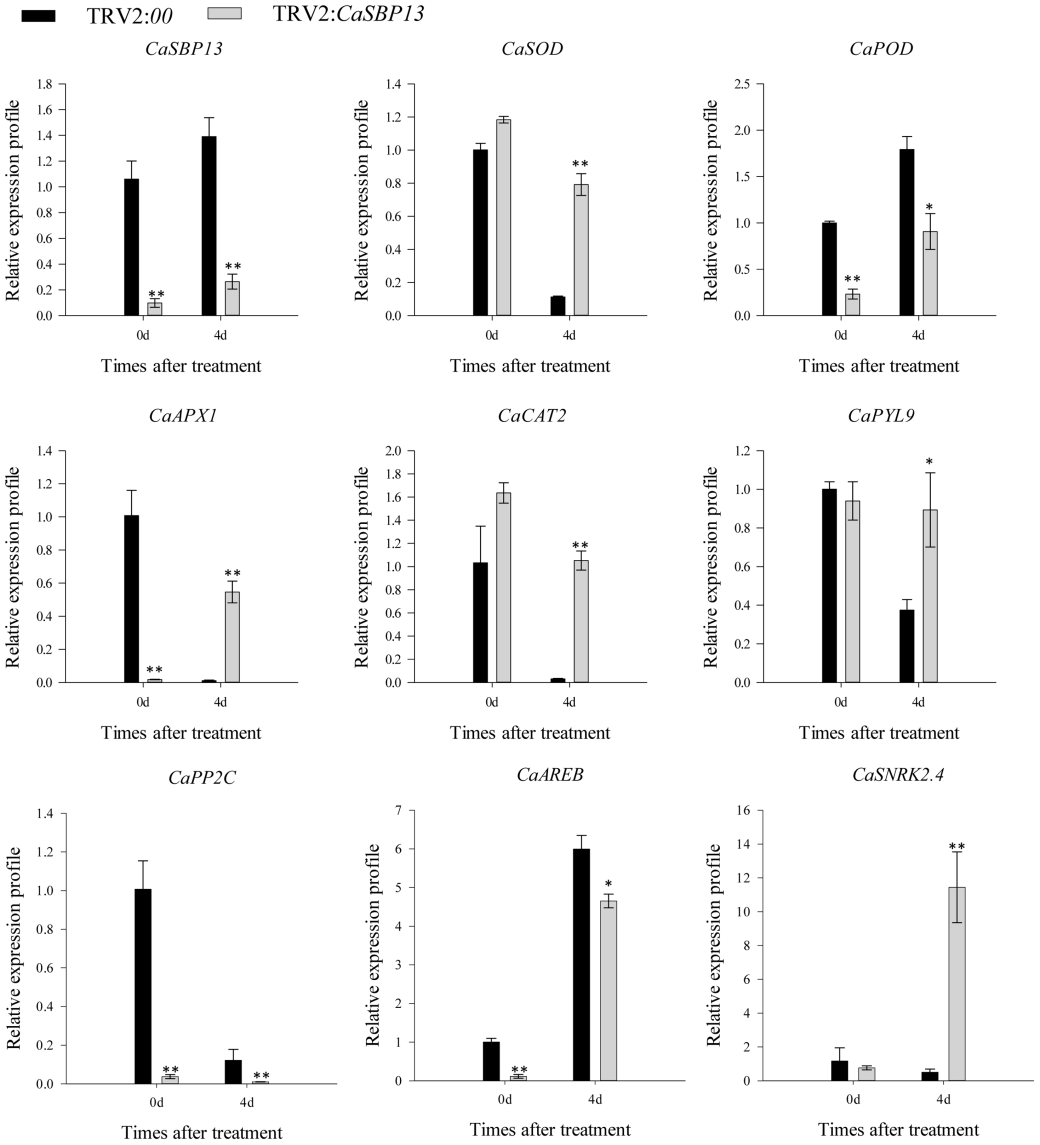
The expression of ROS-scavenging enzymes related genes and the key genes of ABA signaling pathway after drought stress in CaSBP13-silenced and control plants. One-way ANOVA was employed to examine the differences between treatments, significant variances were identified via Tukey’s *post hoc* test. * and ** represent significant differences at *P* ≤ 0.05 and *P* ≤0.01 respectively. Mean values and SDs for three replicates are shown.

To further analyzes, gene expressions of ROS scavenging enzymes (including *CaAPX1*, *CaCAT2*, *CaSOD*, and *CaPOD*) and key genes in ABA signaling pathway modeling (such as *CaPYL9*, *CaPP2C*, *CaAREB*, and *CaSNRK2.4*) were examined, as **Figure 3** illustrates. At Day 4 of drought stress, the expression of *CaPOD* in CaSBP13-silenced plants was significantly lower compared to control, while others (*CaAPX1*, *CaCAT2*, *CaSOD*) were notably higher (**Figure 3**). Moreover, we assessed the expression dynamics of these key genes in the ABA signaling pathway. As suggested in **Figure 3**, following 4 days of drought stress, *CaPYL9* and *CaSNRK2.4* manifested higher expression in CaSBP13-silenced plants, while *CaPP2C* and *CaAREB* displayed lower expression in these plants compared to the control. Significantly, even without treatment, the expression of *CaPOD*, *CaAPX1*, *CaPP2C*, and *CaAREB* expression was higher in the CaSBP13-silenced plants compared to the control plants (**Figure 3**).

### Overexpression of *CaSBP13* notably compromises drought tolerance in *Nicotiana Benthamiana*

**Figure 4:**
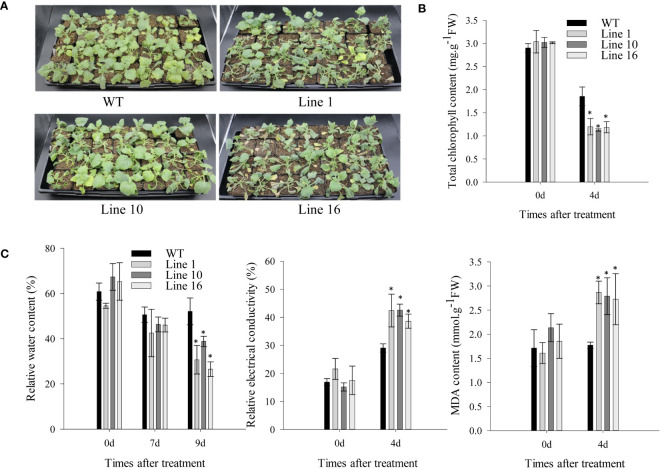
Overexpression of *CaSBP13* in *Nicotiana Benthamiana* enhanced susceptibility to drought stress. **(A)** The phenotype of transgenic and wild-type lines under seven days of drought stress. **(B)** The total chlorophyll content of transgenic and wild-type lines after four days of drought stress. **(C)** The relative water content, relative electrical conductivity, malondialdehyde (MDA) content of transgenic and wild-type lines after four days of drought stress. One-way ANOVA was employed to examine the differences between treatments, significant variances were identified via Tukey’s *post hoc* test. *represent significant differences at *P* ≤ 0.05. Mean values and SDs for three replicates are shown.

**Figure 5:**
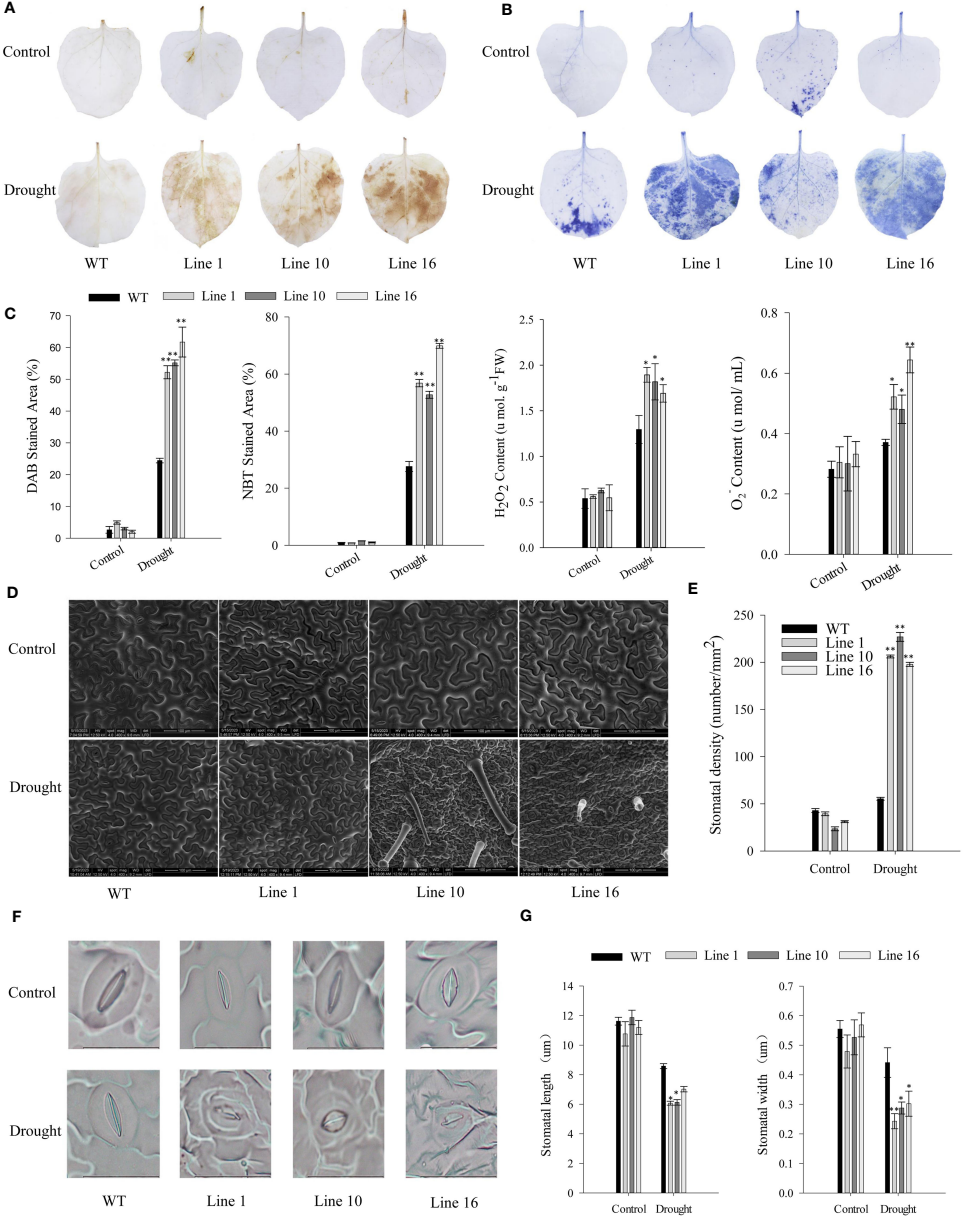
DAB and NBT staining of transgenic and wild-type lines, coupled with quantification of stomatal density. **(A)** DAB staining in leaves of transgenic and wild-type lines after four days of drought stress. **(B)** NBT staining in leaves of transgenic and wild-type lines after four days of drought stress. **(C)** DAB and NBT stained area of transgenic and wild-type lines after four days of drought stress. The H_2_O_2_ and O_2_^-^ content of transgenic and wild-type lines after four days of drought stress. **(D, E)** stomatal density assessment. Scale bar, 100 µm. **(F, G)** The morphological features and length/width of stomata. Scale bar, 20 µm. One-way ANOVA was employed to examine the differences between treatments, significant variances were identified via Tukey’s *post hoc* test. * and ** represent significant differences at *P* ≤ 0.05 and *P* ≤0.01 respectively. Mean values and SDs for three replicates are shown.

To further study the effects of *CaSBP13* on drought resistance, it was overexpressed in *Nicotiana Benthamiana*. Transgenic lines 1, 10, and 16 overexpressing *CaSBP13* were chosen for subsequent analysis. Their expression levels are shown in **Supplementary Figure 3**. Following 7 days of drought, both transgenics and wild-types exhibited wilting (**Figure 4A**). Notably, the lower leaves of the transgenics display severe wilting and yellowing compared to the wild-type (**Figure 4A**). Similarly, at day 4 of drought, the chlorophyll content of the transgenics was markedly diminished compared to the wild-type (**Figure 4B**). Moreover, their relative electrical conductivity and MDA content were higher than the wild-type plants (**Figure 4C**). Over time during drought, the relative water content of the transgenics decreased significantly, reaching a notable decline by day 9 of drought stress (**Figure 4C**). Furthermore, at day 4 of drought, the DAB and NBT staining areas of the transgenics were notably larger than the wild-type plant (**Figures 5A–C**). Correspondingly, the H_2_O_2_ and O_2_^-^ content in the transgenics was drastically higher than the wild-type (**Figure 5C**). Additionally, the stomatal density of the transgenics was significantly elevated compared to the wild-type (**Figures 5D, E**). Besides, both the length and width of stomata in both transgenics plants and wild-type plants decreased, but these variables in the wild-type plants significantly surpassed those in the transgenics plants (**Figures 5F, G**).

**Figure 6:**
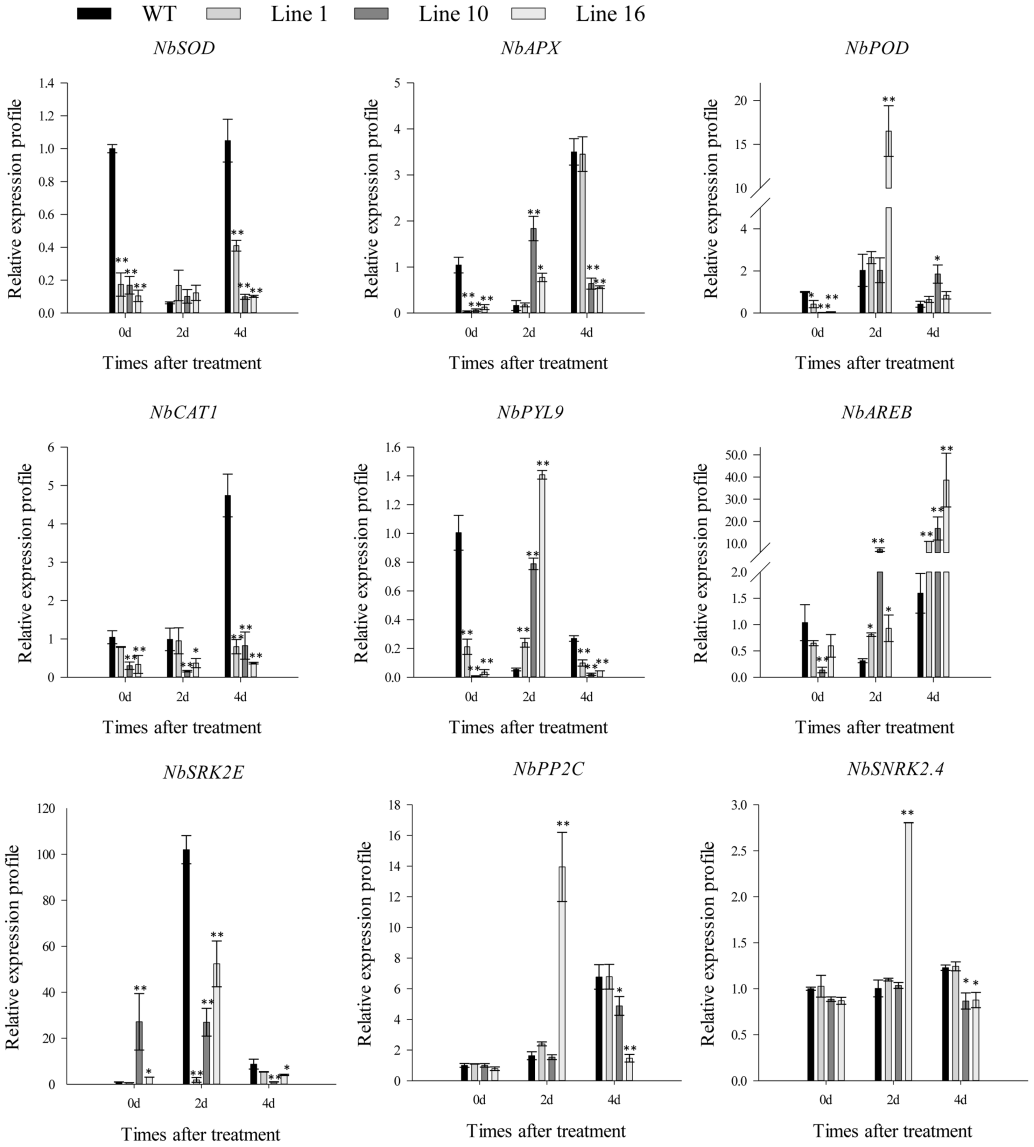
The expression of ROS-scavenging enzymes related genes and the key genes of ABA signaling pathway after drought stress in transgenic and wild-type lines. One-way ANOVA was employed to examine the differences between treatments, significant variances were identified via Tukey’s *post hoc* test. * and ** represent significant differences at *P* ≤ 0.05 and *P* ≤ 0.01 respectively. Mean values and SDs for three replicates are shown.

Based on the above results, we suggest that *CaSBP13’s* function under drought stress may be related to active oxygen and ABA signaling pathways. Therefore, we evaluated the expression of genes involved in active oxygen metabolism and the ABA core pathway, and assessed germination and root growth of the transgenic plants under varying ABA concentration gradients. When subjected to drought stress for four days, the expression of *NbSOD*, *NbAPX*, and *NbCAT1* was significantly lower in the transgenics than in the wild-type (**Figure 6**). Conversely, the expression of *NbPOD* was augmented compared to the wild-type. Of particular note, the expression of critical genes in the ABA signaling pathway model such as *NbPYL9*, *NbSRK2E*, *NbPP2C*, and *NbSNRK2.4* was significantly reduced in the transgenics compared to the wild-type (**Figure 6**). However, the expression of *NbAREB* in the transgenics was notably higher than in the wild-type. Without external intervention, the expression of *NbSOD*, *NbAPX*, *NbPOD*, *NbCAT1*, *NbPYL9*, and *NbAREB* genes was significantly lower in the transgenics than in the wild-type (**Figure 6**). Furthermore, the germination rate of trangenics and wild-type plants declined with increasing ABA concentration. Under 0g/LABA treatment, both types germinated rapidly, achieving full germination by day 5. At 0.1g/LABA, the germination rate of trangenics plants was significantly higher than that of wild-type plants on day 3, achieving full germination by day 5. Similarly, at 0.5g/LABA, the germination rate of trangenics plants was significantly higher than that of wild-type plants on day 3, achieving full germination by day 10. Lastly, under 1g/LABA, the germination rate of trangenics plants was significantly higher than that of wild-type plants on day 5, achieving full germination by day 10 (**Supplementary Table 2**). Additionally, the root length of trangenics and wild plants decreased with increasing treatment concentration in different concentration gradients, but the root length of trangenics plants was significantly higher than that of wild-type plants under 0.1g/LABA, 0.5g/LABA, and 1g/LABA treatments (**Supplementary Figures 4A, B**). Overall, these findings suggest that overexpression of *CaSBP13* in *Nicotiana Benthamiana* exacerbates the vulnerability of the transgenic plants to drought stress.

## Discussion

The SBP-box gene, a specific transcription factor in plants, influences plant growth, signaling, and stress responses. However, its function under drought stress in pepper remains elusive. Here, we demonstrate that one SBP-box gene, *CaSBP13*, suppresses plant defense against drought stress.

The *CaSBP13* amino acid sequence contains all the features of typical SBP-box proteins including two zinc finger-like structures (C3H, C2HC) and a putative nuclear localization signal (Zhang et al., 2016). Furthermore, prior experiments demonstrate that CaSBP13 localizes to the nucleus (Zhang, 2020). Besides, it can be induced by drought stress and suppressed by ABA treatment at 12h (**Supplementary Figure 1**). Additionally, silencing *CaSBP13* enhanced plant tolerance to drought stress (**Figure 1**). It has been reported that under drought conditions, elevated levels of reactive oxygen species (ROS) accumulate in plants, causing oxidative damage to proteins, carbohydrates, lipids and DNA. This initiates an antioxidant defense mechanism, generating enzymes such as catalase (CAT), superoxide dismutase (SOD), and peroxidase (POD) to counteract ROS damage (Gill and Tuteja, 2010). Moderate drought induces stomatal opening, but prolonged drought leads to closure due to ABA stimulation, reducing transpiration (Ashraf, 2010). In this study, it was observed that CAT activities in CaSBP13-silenced plants surpassed those in controls. Additionally, the peak of SOD activity appeared earlier in controls than in CaSBP13-silenced plants (**Figure 1C**). However, the peak of POD activity appeared later and lower in control than in CaSBP13-silenced plants (**Figure 1C**). Remarkably, CaSBP13-silenced plants accumulation of H_2_O_2_ and O_2_^-^ was lower than controls (**Figure 2**). Increased stomatal density was observed in both silenced and control plants, though controls had a higher count (**Figures 2G, H**). Furthermore, post-drought stress, changes in ROS-scavenging enzymes including *CaAPX1*, *CaPOD*, *CaCAT2*, and *CaSOD* were detected in CaSBP13-silenced plants. After 4 days of drought stress, the expression of *CaAPX1*, *CaSOD*, *CaCAT2* was elevated compared to the control, except for *CaPOD* (**Figure 3**). Escalation of *BpSPL9* immune response supported ROS scavenging via peroxidase (POD) and superoxide dismutase (SOD) enzymes, augmenting plant resilience to drought and salt stress (Ning et al., 2017). Overexpression of *OsSPL10* appeared pivotal in drought resilience by controlling ROS generation and stomatal functions (Li et al., 2023). Over-expression of *TaSPL6-A* decreased wheat drought resilience, signifying unrestrained ROS elevation (Zhao et al., 2024). Augmentation of the ‘SiJiMi’ mango gene *MiSPL3a/b* in *Arabidopsis* enhanced drought resistance in transgenics (Zhu et al., 2024). Identifying higher stomatal densities in the CaSBP13-silenced plants, we suggest the role of drought tolerance by *CaSBP13* may encompass the ABA signaling pathway. Consequently, expression elevations of *CaPYL9*, and *CaSNRK2.4* were observed in CaSBP13-silenced plants post drought stress (**Figure 3**). Conversely, expressions of *CaPP2C*, and *CaAREB* were reduced in CaSBP13-silenced plants compared to control plants. Notably, the expression of *CaPOD*, *CaAPX1*, *CaPP2C*, and *CaAREB* was markedly lessened in CaSBP13-silenced plants under normal conditions, suggesting a regulatory role for *CaSBP13* during drought stress. In the ABA signal pathway, there are three critical components: *PYL*, an ABA receptor, *PP2C*, a key negative regulator, and *SnRK2*, a significant positive mediator. These components establish a complex interplay to manipulate ABA signal transduction and responses, forming a dual negative regulatory system (PYL-PP2C-SnRK2) (Sun et al., 2020). Under non-stressful conditions, where ABA levels are restricted, *PYL* does not bind to ABA. *PP2C* suppresses the activation of *SnRK2* by dephosphorylation, halting the activation of transcription factors *ABF/AREB* upon ABA perception, resulting in pathway inactivation. However, under severe conditions such as drought, salinity or elevated temperature, the fast accumulation of plant ABA incites the formation of a PYL-ABA complex, initiating intermolecular interactions between PYL and PP2C. This impedes dephosphorylation of *SnRK2* by *PP2C*, thereby releasing the suppression of *PP2C* on *SnRK2*. Phosphorylated *SnRK2* subsequently stimulates or suppresses the expression of various downstream transcriptional regulators or effectors, initiating ABA signal transduction and regulating plant growth, development, or stress response (Cutler et al., 2010; Zhao et al., 2013). Besides, it has been reported that phosphorylation of *CaNAC035* by *CaSnRK2.4* modulates abscisic acid synthesis in pepper under cold stress (Zhang et al., 2023). To validate *CaSBP13’s* role in plant drought stress response, *N. benthamiana* overexpressing *CaSBP13* was engineered. These transgenic plants exhibited heightened susceptibility to drought stress, exhibiting increased relative electrical conductivity, MDA concentration, and active oxygen accumulation (**Figures 4**, **5**). Notably, the transgenic plants had significantly reduced expression of *NbSOD*, *NbAPX*, and *NbCAT1* compared to wild-type plants post-4 days of drought stress, mirroring previous pepper research findings (**Figures 3**, **6**). Correspondingly, the levels of *NbPYL9* and *NbSNRK2.4* in the transgenic plants were diminished at day 4 compared to wild type plants, along with a marked upregulation of *NbAREB*, corroborating predictions from pepper studies (**Figures 3**, **6**). In contrast, without any treatments conditions, *NbSOD*, *NbAPX*, *NbPOD*, *NbCAT1*, *NbAREB*, and *NbPYL9* gene expressions in the transgenic plants were notably lower than wild type plants (**Figure 6**). Moreover, both the germination rate and root length of the transgenic and wild-type plants were suppressed under varying ABA concentrations. However, the wild-type plants exhibited a greater response to ABA treatment than the transgenic plants(**Supplementary Table 2**, **Supplementary Figure 4**). Therefore, we propose that the *CaSBP13* gene enhances plant drought resistance via ROS and ABA signaling pathways. Nevertheless, further empirical validation is necessary to confirm these proposals.

## Conclusions

In conclusion, the expression of *CaSBP13* gene rises during drought stress in pepper. Silencing *CaSBP13* improves plants drought resistance witch lowering ROS production compared to control plants. However, *CaSBP13* overexpression in *N. benthamiana* intensifies drought sensitivity and ROS production in comparison to wild-type plants. Four days post drought stress, *CaAPX1*, *CaCAT2*, and *CaSOD* expression is heightened in CaSBP13-silenced plants compared to controls, while *CaPOD* expression is reduced. Also, *NbCAT1*, *NbSOD* and *NbCAT1* transcripts decrease while *NbPOD* transcription increases in CaSBP13-overexpressiing plants. Remarkably, under nonstressed conditions, *CaPP2C*, and *CaAREB* transcripts of the ABA signaling pathway are significantly reduced in CaSBP13-silenced plants. Similarly, *NbAREB*, and *NbPYL9* transcripts of the ABA signaling pathway are lower in transgenics than wild-types plants under similar conditions. These findings suggest a negative effect of *CaSBP13* on plant drought tolerance possibly linked to its role in the ROS- and ABA-signaling pathways. Additional experimentation is needed to elucidate these mechanisms.

## Data availability statement

The original contributions presented in the study are included in the article/**Supplementary Material**. Further inquiries can be directed to the corresponding author.

## Author contributions

H-XZ: Conceptualization, Data curation, Formal analysis, Funding acquisition, Investigation, Methodology, Project administration, Resources, Supervision, Validation, Visualization, Writing – original draft, Writing – review & editing. YZ: Data curation, Formal analysis, Methodology, Writing – review & editing. B-WZ: Data curation, Methodology, Software, Writing – review & editing.
