# Supplementary material for: Pepper SBP-box transcription factor, CaSBP13, plays a negatively role in drought response
Source: Front Plant Sci. 2024 Jul 12;15:1412685. doi: 10.3389/fpls.2024.1412685 (PMC11272568; doi:10.3389/fpls.2024.1412685)

**SUPPLEMENTARY TABLES AND FIGURES**

**Supplementary Figure 1.** The expression profile of *CaSBP13* gene under drought and ABA stress in pepper. (A) After 20% Polyethylene glycol (PEG6000) treatment, the expression of *CaSBP13* was detected at 0h, 3h, 6h, 9h, 12h, and 24h. h: hour.(B) After 20μM ABA treatment, the expression of *CaSBP13* was detected at 0h, 3h, 6h, 9h, 12h, 24h, and 48h. One-way ANOVA was employed to examine the differences between treatments, significant variances were identified via Tukey’s post hoc test. * and ** represent significant differences at *P* ≤ 0.05 and *P* ≤0.01 respectively. Mean values and SDs for three replicates are shown.

**
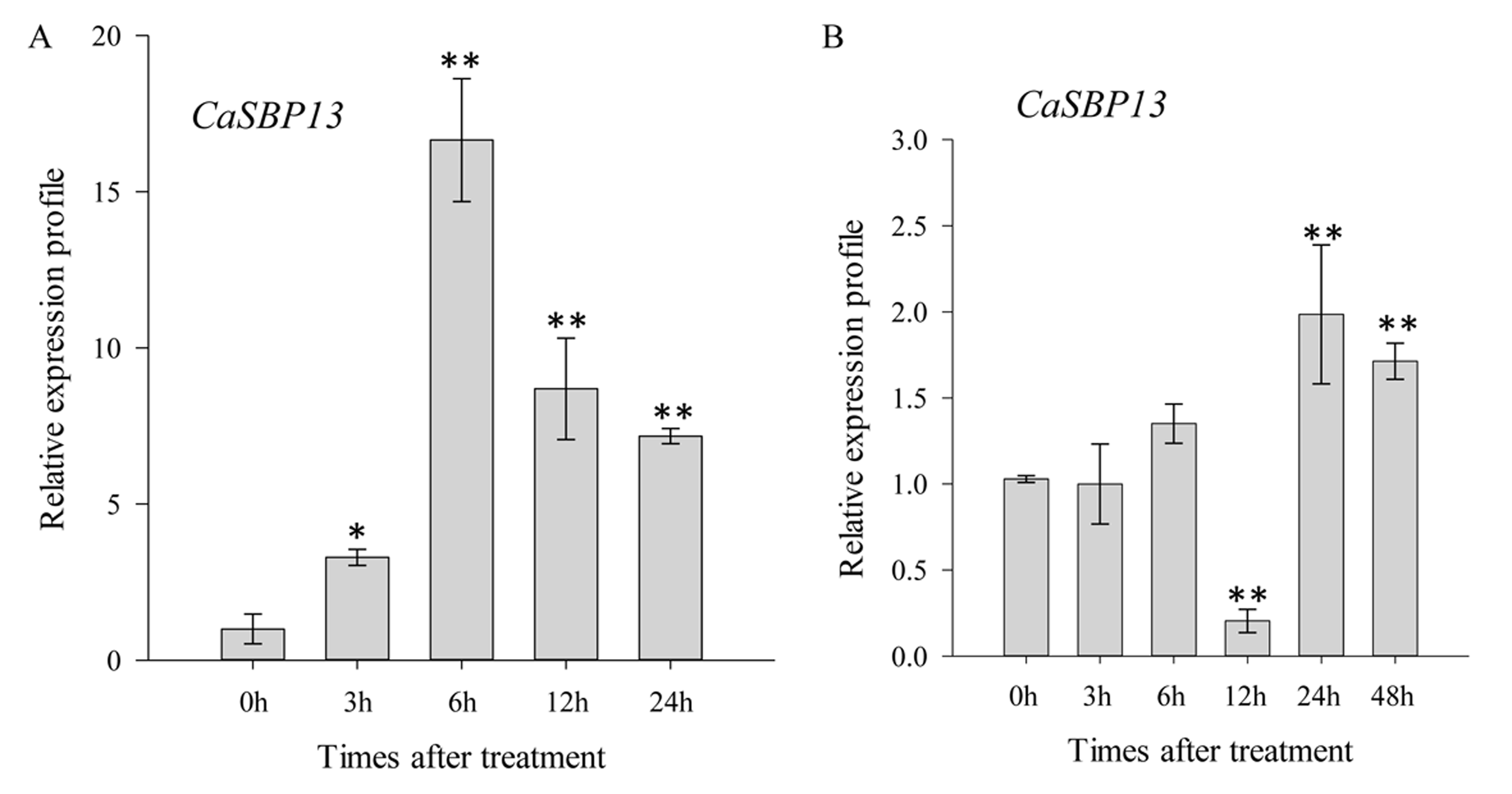
**

**Supplementary Figure 2.** Phenotype and silencing efficiency of CaSBP13- silenced plant. (A) Phenotype of CaSBP13- silenced plant. Photographs were taken forty days after injection. The diameter of the pot is 7 cm. (B) The silencing efficiency of *CaSBP13* in the silenced and negative control plants. One-way ANOVA was employed to examine the differences between treatments, significant variances were identified via Tukey’s post hoc test. ** represents significant differences at *P* ≤0.01. Mean values and SDs for three replicates are shown.

**
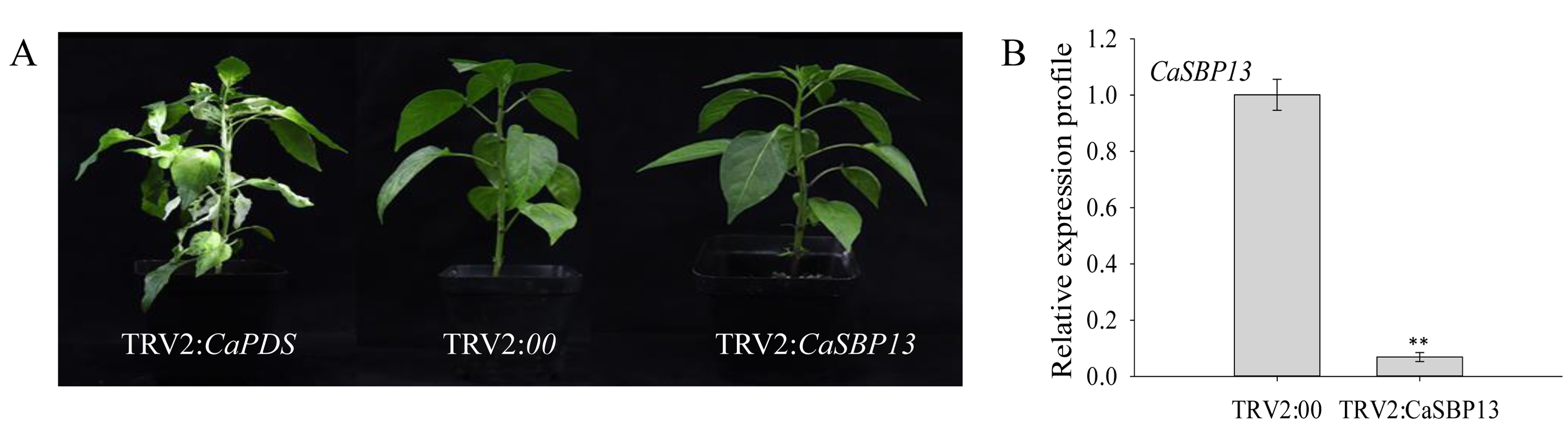
**

**Supplementary Figure 3.** The expression of *CaSBP13* gene in transgenic lines and wild-type lines of *Nicotiana benthamiana*. One-way ANOVA was employed to examine the differences between treatments, significant variances were identified via Tukey’s post hoc test. ** represent significant differences at *P* ≤ 0.01.Mean values and SDs for three replicates are shown.


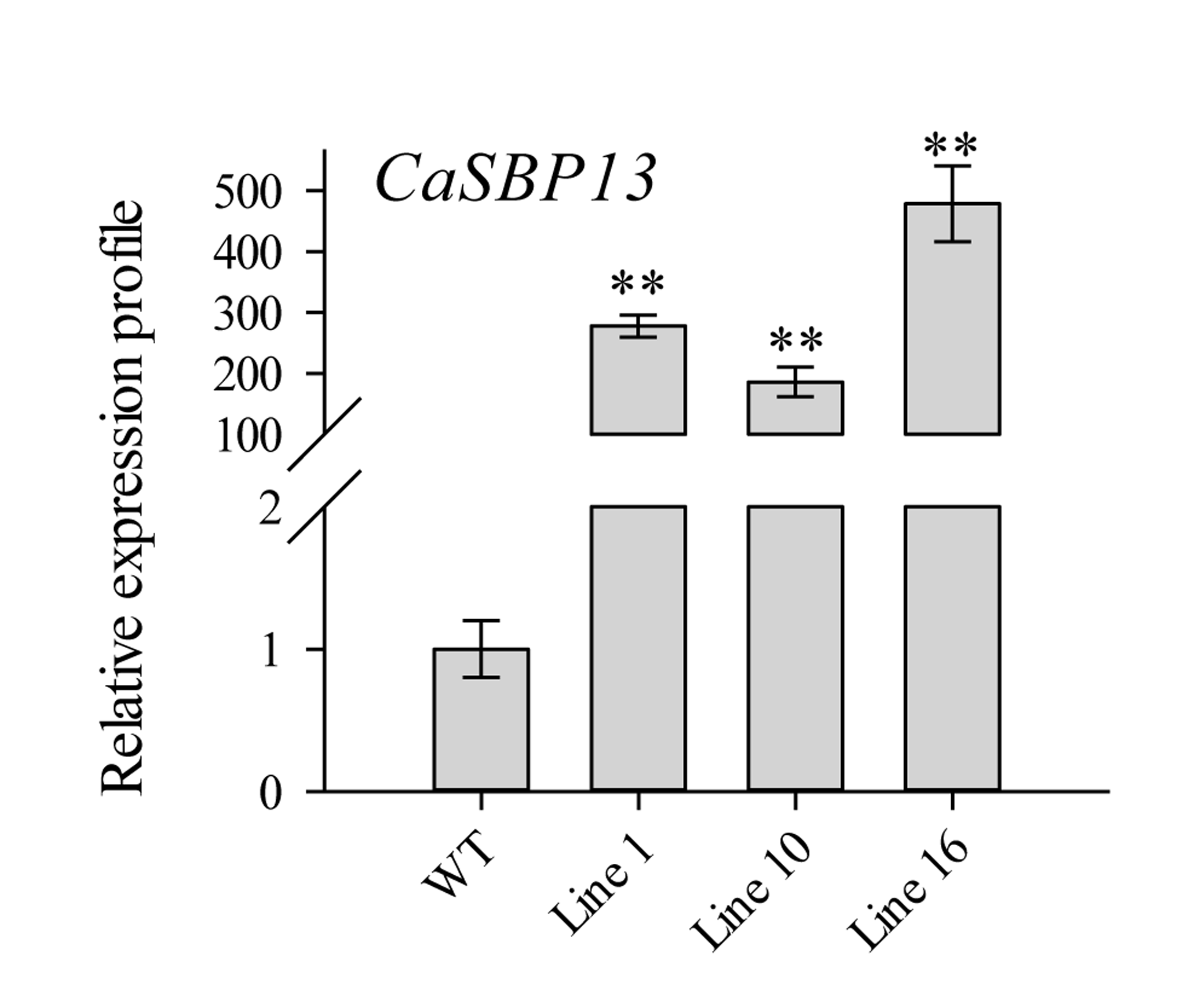


**Supplementary Table 1.** Primers names and their sequences used for vector construction and quantitative PCR.

| **Oligo Name** | **Primer Abbreviation** | **Primer Sequence (5^’-^3’)** |
| --- | --- | --- |
| ***CaSBP13*** | CaSBP13-VIGS-F | CGGGATCCTGTTTTCCTGGAGTCTCAGATTC |
|  | CaSBP13-VIGS-R | GGGGTACCCATCGCCATGCTGAGCTATC |
|  | CaSBP13-2307-GFP-F | GCTCTAGAATGGGCACTTGGAAGAAGTCTG |
|  | CaSBP13-2307-GFP-R | GGGGTACCAAGAGTCCAGTGCACATTCTGAAC |
| ***CaUBI3*** | CaUBI3-F | TGTCCATCTGCTCTCTGTTG |
|  | CaUBI3-R | CACCCCAAGCACAATAAGAC |
| ***CaAPX1*** | CaAPX1-F | AGAGGACAAGCCAGAACCAC |
|  | CaAPX1-R | CCTTGTCTGATGGCAACTGT |
| ***CaCAT2*** | CaCAT2-F | GAAGCCAAATCCTAAGTCCC |
|  | CaCAT2-R | CCAACTCGGATTGCCTCTT |
| ***CaSOD*** | CaSOD-F | TATGGAGCCTTAGAACCTGC |
|  | CaSOD-R | CCATTGAACTTGATAGCACCT |
| ***CaPOD*** | CaPOD-F | TCCTCCTCCTACTTCTAACC |
|  | CaPOD-R | ACAGACCTCTTTTGCTCACT |
| ***CaPYL9*** | CaPYL9-F | CGTTGAAAGGAGGAGT |
|  | CaPYL9-R | ACAAGATGAACAGGAGC |
| ***CaPP2C*** | CaPP2C-F | GTGATTCTCGTGCTGTGC |
|  | CaPP2C-R | CAGGCAGTCTCGTTTGAT |
| ***CaAREB*** | CaAREB-F | GGAAATGAGCCACCAG |
|  | CaAREB-R | CCCACCAATCCCAGTA |
| ***CaSNRK2.4*** | CaSNRK2.4-F | TGAGAATGTAGCAAGGGAG |
|  | CaSNRK2.4-R | GCAACAGGGACGACTT |
| ***Nbactin-97*** | Nbactin-F | TATGGAAACATTGTGCTCAGTGG |
|  | Nbactin-R | CCAGATTCGTCATACTCTGCC |
| ***NbAPX*** | NbAPX-F | CCAAGGGTTCTGACCATCTG |
|  | NbAPX-R | GCATAGTCGGCAAAGAAAGC |
| ***NbCAT1*** | NbCAT1-RT-F | TCTATTGTGGTTCCAGGGGTTT |
|  | NbCAT-RT-R | CACCCACCGACGAATAAAGC |
| ***NbSOD*** | NbSOD-RT-F | GCAGACGGACCTTAGCAACA |
|  | NbSOD-RT-R | TGGCGACGGTAGGAGCAT |
| ***NbPOD*** | NbPOD-RT-F | AGGCTCAGGGGACAACAACT |
|  | NbPOD-RT-R | TCACAAAATCAGTGGCGAAA |
| ***NbPYL9*** | NbPYL9-F | ACCGTTTGTTAGCAGG |
|  | NbPYL9-R | CGTCTTTAGTATTCCCATCT |
| ***NbPP2C*** | NbPP2C-F | TGTCGGAATCTGATGTTT |
|  | NbPP2C-R | ACGGGTGAACTGCTACT |
| ***NbAREB*** | NbAREB-F | TTTCGTTCAAGGTGCTG |
|  | NbAREB-R | CATTCTCCTTTGCCTCC |
| ***NbSNRK2.4*** | NbSNRK2.4-F | TTCCCGTTCAGTCTCAGGCTT |
|  | NbSNRK2.4-R | TGTGCCTGCTTCACTTGCTTT |
| ***NbSRK2E*** | NbSRK2E-F | TCTAAGTCCTCGGTGTTG |
|  | NbSRK2E-R | TTCGCTGTATTGTCTTGC |

**Supplementary Table 2.** Germination rate of seeds under ABA treatment.

| Time | Plants | | 0g/LABA | | --- | | 0.1g/LABA | 0.5g/LABA | 1g/LABA |
| --- | --- | --- | --- | --- | --- |
| 3d | WT | 0.989±0.019a | 0.533±0.053b | 0.101±0.089c | 0.000±0.000b |
|  | Line 1 | 0.963±0.088a | 0.822±0.051a | 0.301±0.058b | 0.000±0.000b |
|  | Line 10 | 0.967±0.041a | 0.901±0.011a | 0.211±0.056b | 0.000±0.000b |
|  | Line 16 | 0.944±0.051a | 0.889±0.052a | 0.756±0.086a | 0.101±0.011a |
| 5d | WT | 1.000±0.000a | 1.000±0.000a | 0.922±0.077a | 0.589±0.068b |
|  | Line 1 | 0.991±0.051a | 1.000±0.000a | 0.978±0.038a | 0.811±0.019a |
|  | Line 10 | 1.000±0.000a | 1.000±0.000a | 0.911±0.038a | 0.722±0.051a |
|  | Line 16 | 1.000±0.000a | 1.000±0.000a | 0.867±0.058a | 0.070±0.067a |
| 10d | WT | 1.000±0.000a | 1.000±0.000a | 1.000±0.000a | 1.000±0.000a |
|  | Line 1 | 1.000±0.000a | 1.000±0.000a | 1.000±0.000a | 1.000±0.000a |
|  | Line 10 | 1.000±0.000a | 1.000±0.000a | 1.000±0.000a | 1.000±0.000a |
|  | Line 16 | 1.000±0.000a | 1.000±0.000a | 1.000±0.000a | 1.000±0.000a |

One-way ANOVA was employed to examine the differences between treatments, significant variances were identified via Tukey’s post hoc test. Letters indicate significant differences at *P* ≤ 0.05.

**Supplementary Figure 4.** The root length of transgenic and wild-type plants under diverse ABA treatments. (A) The morphological assessment of transgenic and wild-type plants across 10 days after treatments with distinct ABA concentrations. (B) The root length of transgenic and wild-type plants, after 10 days of treatment with varying ABA concentrations. One-way ANOVA was employed to examine the differences between treatments, significant variances were identified via Tukey’s post hoc test. * represent significant differences at *P* ≤ 0.05. Mean values and SDs for three replicates are shown.


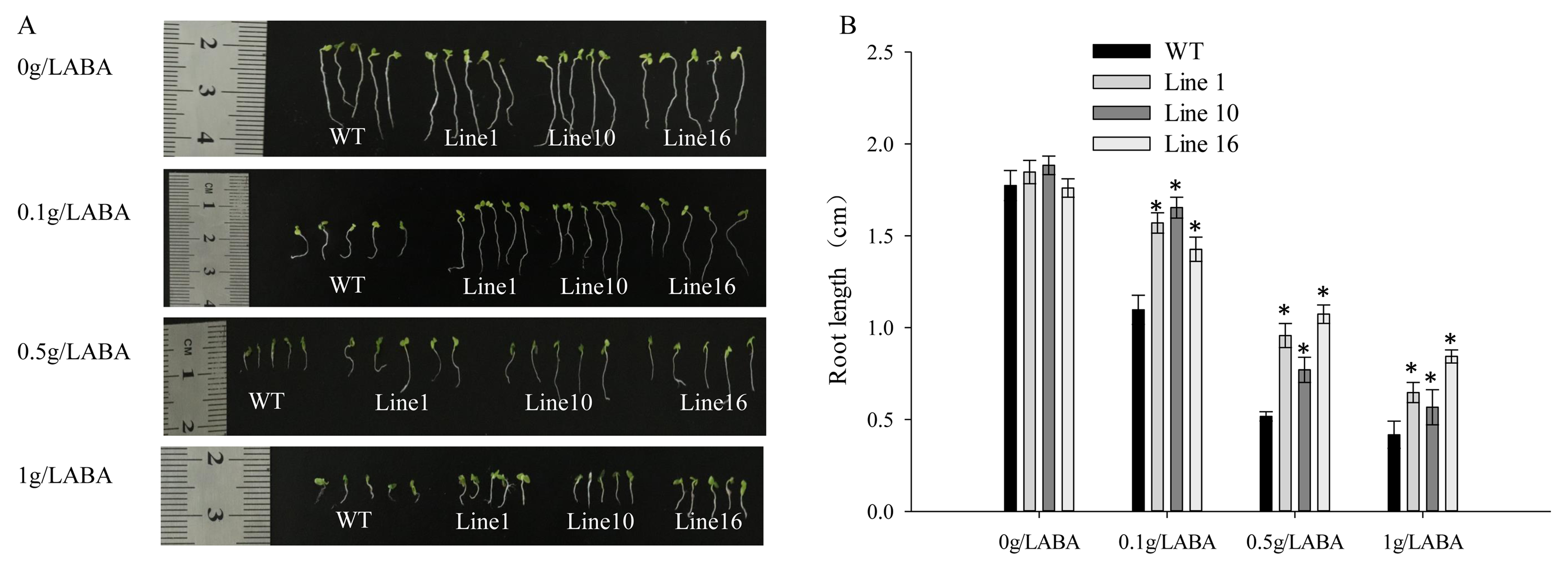

Supplement: Supplementary file 1 [file DataSheet_1.doc]
